# The Management of Large Bladder Calculi by Utilizing Dual-Action Percutaneous Lithotripsy via Suprapubic Tube Sheath: A Novel Technique

**DOI:** 10.7759/cureus.56326

**Published:** 2024-03-17

**Authors:** Kayla G Meyer, Kristofer Muzzi, Nicole Ambrose, Zachary Snow, Zachariah Taylor, Teodora Schellato, Noah R May

**Affiliations:** 1 Urology, Philadelphia College of Osteopathic Medicine, Philadelphia, USA; 2 Urology, Main Line Health, Bryn Mawr, USA; 3 Urology, MidLantic Urology, Philadelphia, USA

**Keywords:** urolithiasis, endourology, endoscopic surgery, percutaneous lithotripsy, bladder calculi

## Abstract

Bladder calculi commonly develop in patients with poor bladder emptying or those with retained foreign bodies within the bladder, leading to irritative voiding symptoms, hematuria, and an increased likelihood of refractory urinary tract infections. While many techniques exist for the treatment of bladder calculi, including endoscopic and open-surgical approaches, our novel technique may help manage exceptionally large or difficult-to-treat bladder calculi effectively. We present three patients with symptomatic bladder calculi ranging from 1.3 cm to 6.8 cm in size who were successfully treated by using our novel technique. Percutaneous access to the bladder was obtained by using a suprapubic catheter trocar and sheath to enable the utilization of a dual-action lithotriptor. Sheath insertion and lithotripsy were performed under direct visualization with a rigid cystoscope via the native urethra. This technique is easily learned and can be safely employed in patients in whom other methods may pose risks of higher morbidity.

## Introduction

Urolithiasis is a common health issue resulting in a significant burden to the healthcare system, with bladder calculi accounting for approximately 5% of urinary stones [1,2]. Traditionally, bladder calculi have been managed with either an open approach or cystolitholapaxy [3]. However, for large bladder calculi, open cystolithotomy remains the mainstay of treatment. Minimally invasive endoscopic and percutaneous approaches, which offer the benefits of shorter duration of catheterization, hospital stay, operative time, and recovery compared to open surgery, have gained in popularity [4,5]. Among these approaches, using a nephroscope (either per urethra or via a percutaneous approach) has demonstrated shorter procedure duration times compared to laser lithotripsy, thanks to the utilization of a dual-action lithotriptor [6]. However, the use of a nephroscope via the urethra may be challenging due to its large diameter. Additionally, nephroscope use may result in a large bladder defect if placed percutaneously [7]. We present three cases where a novel technique was employed to remove bladder calculi safely and effectively by using a dual-action pulse lithotripter via percutaneous access in conjunction with retrograde cystoscopy.

## Case presentation

Technique

The patients received general anesthesia and were placed in the dorsal lithotomy position. A 21 French (F) cystoscope was used to perform cystoscopy. If a new suprapubic trocar was needed, the suprapubic tract was anesthetized, and a 16F suprapubic tube (SPT) trocar was placed under direct visualization (Rusch). The introducer trocar was removed, leaving the 16F sheath, and the breakaway sheath tab was then cut off. A standard cystoscope port rubber nipple was placed on the sheath to prevent irrigation fluid leakage. The trocar was inserted through the previous SPT tract for patients with a preexisting SPT. A dual-action lithotriptor (Olympus Shockpulse for patients 1 and 2, Boston Scientific Trilogy for patient 3) was then operated via the SPT sheath to fragment and evacuate the stone. Visualization was maintained by the gravity flow of normal saline through the cystoscope. The cystoscope sheath was upsized to 23F or 25F as possible to maximize stone fragment removal. Cystoscopic evaluation of the bladder was performed to ensure stone-free status. Once the stone was cleared, a 16F foley was placed via the SPT sheath and maintained for 1-2 weeks. The patient and operative case characteristics are summarized in Table 1.

**Table 1:** Patient and operative characteristics

Characteristic	Case 1	Case 2	Case 3
Age	60 years	71 years	72 years
Gender	Male	Male	Male
Stone size	6.8 cm	1.3 cm, 3.8 cm	3.4 cm
Number of stones	1	2	1
Stone-free	Yes	Yes	Yes
Estimated blood loss	None	None	None
Operative time	260 minutes	60 minutes	85 minutes
Postoperative complications	None	None	None

Case 1

A 60-year-old quadriplegic male with chronic SPT was found to have a 6.8 cm bladder calculus. Given his obese body habitus and difficulty with positioning, our novel technique utilized his existing SPT tract. The case took four hours and 20 minutes. There were no postoperative complications.

Case 2 

A 71-year-old male with a history of prostate cancer and incomplete emptying was found to have two bladder calculi measuring 3.8 cm and 1.3 cm. He underwent percutaneous suprapubic cystolithotomy technique as described through a newly created SPT tract. Each stone was found to contain a surgical clip in its center, presumably from his previous radical prostatectomy. The procedure took 60 minutes, and a SPT was left postoperatively. There were no postoperative complications.

Case 3

A 72-year-old male with incomplete bladder emptying was found to have a 3.4 cm bladder calculus. Initially, he underwent cystoscopy with laser lithotripsy using a thulium laser, which was unsuccessful due to the density of the stone. The patient then underwent percutaneous suprapubic cystolithotomy, as previously described, via a newly created SPT tract. The operative time was 85 minutes. There were no postoperative complications.

## Discussion

**Figure 1:**
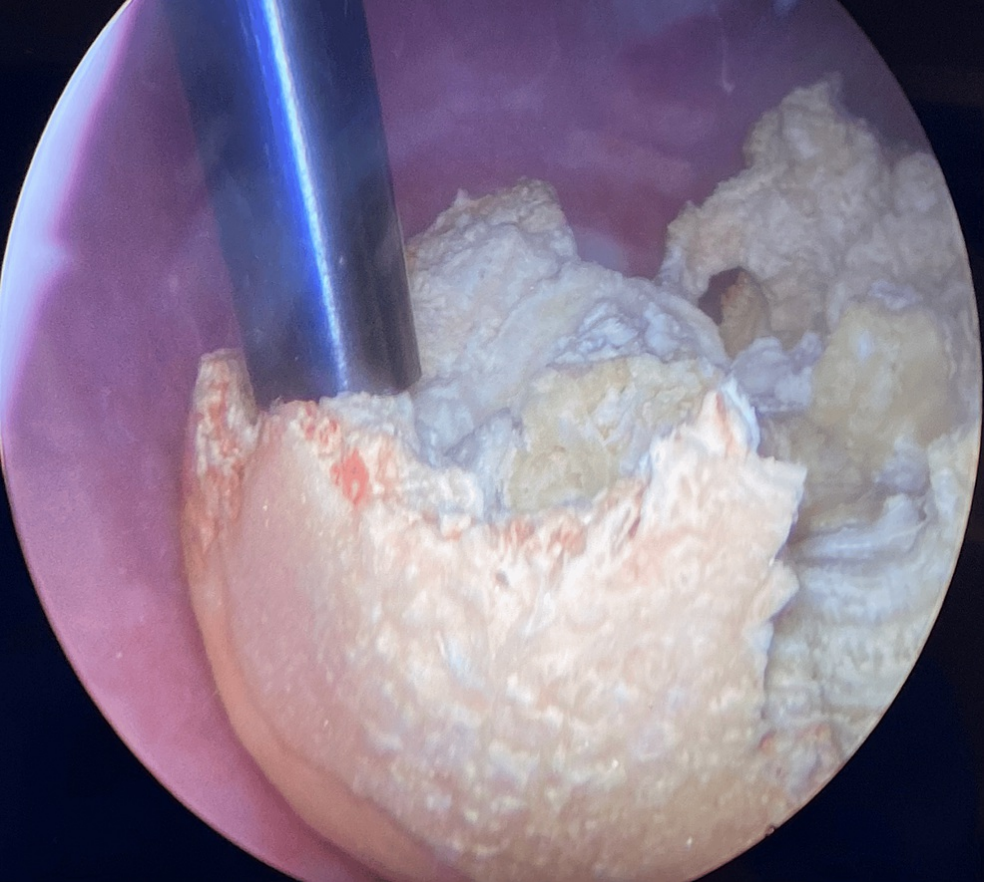
Representative image of the dual-action lithotriptor being visualized from the cystoscope

Bladder calculi represent a common medical condition that can pose treatment-related challenges. Our study discusses three successful cases where a novel technique was utilized (Figure 1) for treating large bladder calculi; this method is especially helpful in individuals unable to undergo an alternative procedure without increased morbidity or significant technical challenges. While additional data is needed to evaluate this novel technique further, we believe that utilizing a 16F SPT sheath for passage of a percutaneous dual-action lithotripter with simultaneous use of a smaller French cystoscope, as opposed to a larger nephroscope, could benefit many patients. Open cystolithotomy often requires a large cystotomy to remove the stone, resulting in postoperative pain, prolonged healing, possible urine leak, and often additional imaging (e.g., cystogram to confirm bladder integrity before catheter removal). Using our method, large stones can be removed with high efficiency and minimal morbidity, allowing for same-day discharge.

Cases 1 and 3 in our study demonstrate two distinct benefits. In case 1, we were able to safely and completely remove an exceptionally large (6.8 cm) bladder calculus in one operation through an existing tract in a patient with significant comorbidities. In case 3, a thulium laser lithotripsy was attempted but failed due to the hardness of the stone. However, the dual-action lithotriptor efficiently fragmented and evacuated the stone.

Multiple options currently exist for the treatment of bladder calculi. The European Association of Urology currently offers stone interventions, including open cystolithotomy, transurethral cystolithotomy, suprapubic cystolithotomy, and laparoscopic techniques. Previous studies have demonstrated that when compared to percutaneous cystolithotripsy, transurethral techniques can have equivalent stone-free rates, especially when a nephroscope is utilized [3]. However, there is scarce data regarding the long-term consequences of the urethral approach, particularly stricture rates. Some studies have reported a urethral stricture rate between 2.9% and 19.6% [8]. Furthermore, the utilization of a larger nephroscope would likely increase the risk of urethral stricture formation. Our technique may benefit patients as it uses a smaller cystoscope sheath, thereby reducing the risk of urethral strictures while still using the dual-action lithotripter.

Clinicians should be mindful of common complications and contraindications, similar to those for percutaneous SPT placement. Contraindications are relatively few. Absolute contraindications include a history of bladder cancer or the inability to adequately distend the bladder. Relative contraindications for the suprapubic approach would include active skin infection, coagulopathy, osteomyelitis of the pubis, or a history of orthopedic hardware in the pubis. Axial imaging should be obtained before the procedure to confirm the absence of bowel anterior to the bladder, which would complicate the placement of the SPT. Furthermore, it may be difficult to safely place the SPT sheath in individuals with a history of pelvic radiation, inflatable penile prosthesis/artificial urinary sphincter reservoirs, or contracted, poorly compliant bladders.

One limitation of the technique is that it generally requires two people to perform the procedure: one to operate the lithotriptor and one to maintain visualization with the cystoscope. Utmost care must be taken to ensure the sheath's position, preventing direct contact of the lithotriptor shaft with the patient’s skin to avoid thermal injury. In our cases, we maintained the SPT for one to two weeks to allow for a tract to form before removing it and to prevent immediate leakage. In patients with intact bladder function, the catheter can be capped at the end of the procedure, eliminating the need for a urethral catheter. Patients with chronic suprapubic catheters can resume regular SPT changes with minimal disruption to their daily routine.

## Conclusions

The management of large bladder calculi by utilizing percutaneous access via a 16F SPT sheath is a highly effective technique that may prove beneficial in many cases, particularly those with large bladder calculi that might otherwise require invasive surgery with prolonged hospital stays. Further research is required to gain deeper insights into the efficiency and safety of the procedure.
